# Ensemble‐based adaptive soft sensor for fault‐tolerant biomass monitoring

**DOI:** 10.1002/elsc.202100091

**Published:** 2022-01-08

**Authors:** Manuel Siegl, Vincent Brunner, Dominik Geier, Thomas Becker

**Affiliations:** ^1^ Chair of Brewing and Beverage Technology Technical University of Munich Freising Germany

**Keywords:** adaptive modeling, biomass prediction, ensemble‐based method, fault tolerance, soft sensor

## Abstract

The accuracy and precision of soft sensors depend strongly on the reliability of underlying model inputs. These inputs (particularly readings of hardware sensors) are frequently subject to faults. This study aims to develop an adaptive soft sensor capable of reliable and robust biomass concentration predictions in the presence of faulty model inputs for a *Pichia pastoris* bioprocess. Hence, three soft sensor submodels were developed based on three independent model inputs (base addition, CO_2_ production, and mid‐infrared spectrum). An ensemble‐based algorithm combined the submodels to form an ensemble model, that is, an adaptive soft sensor, to achieve fault‐tolerant prediction. The algorithm's basic steps are as follows: the initial determination of submodel reliability is followed by selecting appropriate submodels to generate a reliable prediction via variance‐based weighting of the submodels. The adaptive soft sensor demonstrated high robustness and accuracy in biomass prediction in the presence of multiple simulated sensor faults (RMSE = 0.43 g L^−1^) and multiple real sensor faults (RMSE = 0.70 g L^−1^).

**Abbreviations**
CER: carbon dioxide emission rate
MIR: mid‐infrared
PLS: partial least squares
RMSE: root mean square error

## 1 INTRODUCTION

Monitoring biological processes in many cases involves process variables that cannot be measured reliably online or only with delay due to time‐consuming laboratory analyses. However, these variables are often important indicators for assessing the overall process status. Soft sensors can be developed and used to provide real‐time variables. With soft sensors, indirect determination of target variables, also known as primary variables, can be realized using continuously measured process variables, along with process knowledge and statistical analysis. These process variables can be measured in real‐time and are referred to as secondary variables. To predict the primary variables, different soft sensor models are distinguished that combine the secondary variables: Data‐driven, mechanistic, and hybrid models.

Biomass concentration represents a key variable in bioprocesses, for which direct determination is time‐consuming. Typical measurements include counting colonies on agar plates or determining the dry cell weight, both of which take several days due to incubation and drying periods. However, several secondary variables are linked to biomass concentration and can be used for prediction in soft sensor models. Existing literature suggests the online measurement of turbidity [1], infrared [2, 3], or fluorescence spectra [4], as well as O_2_ and CO_2_ concentration in the exhaust gas [5, 6]. Furthermore, actuator information such as the addition of pH correction agents [6] can be suitable, as well.

Nonetheless, online measurements of these secondary variables are frequently subject to interference and faults caused by damaged sensors, connection problems, and insufficient calibration [7]. Common sensor fault types can be classified as bias (intermittent, stepwise, drift‐wise, or cyclic deviation), precision degradation, and temporary or complete sensor failure [8, 9]. These disturbances consequently reduce the prediction performance of soft sensors. Adaptive soft sensors are required to guarantee reliable predictions despite these conditions. The adaptability to disturbances and sensor faults is also known as fault tolerance.

The basic structure of a fault‐tolerant system can be divided into two sections: fault detection and fault compensation. One method for detecting faults is multivariate statistical process control. Process corridors are established by unfolding the historical data matrix of secondary variables and subsequent principal component analysis. By building process corridors, this method is suitable for detecting sensor faults [10], but the actual purpose is rather to detect process faults, i.e., untypical process sequences. Thus, sensor faults can only be detected reliably if the process deviation can be excluded. Another option is the use of symptom signal methods. Here sensor signals are directly compared with an estimate of themselves [11]. A well‐known representative is the Kalman filter. With this technique, process knowledge, such as physical and chemical relationships, can be combined with sensor data through mechanistic models. Thus, detecting and compensating sensor faults using the variance of inputs is possible [12]. However, faulty measurements are not excluded but weighted less. This method has already improved the prediction performance of soft sensors in biological processes [13, 14, 15]. Especially for bioprocesses in defined media, mechanistic models can be used [16], but transferability to complex nonlinear bioprocesses can be challenging [17]. Another way to develop a symptom signal method is based on artificial neural networks (ANNs). Huang et al. [18] developed an artificial auto‐associative neural network (AANN) to detect faulty sensor readings of secondary variables in bioprocesses. In the study, all real‐time measured secondary variables of a bioprocess served as input for the AANN. An estimate of all secondary variables could be retrieved as output after passing through the AANN. However, due to the structure of the AANN, information from all inputs was present in each output. By comparing the output and input layers, sensor faults in secondary variables could be detected using an evaluation index. Nonetheless, the interpretation of neural networks is difficult (black box model) and can lead to problems, especially when directly transferred to other processes. Brunner et al. [19] used another symptom signal to detect sensor faults of secondary variables in a batch process. They used an indicator variable [20] to account for time‐variant behavior and process lengths. The estimation for deriving the symptom signal is determined via the binary particle swarm optimization algorithm and used for fault detection within turbidity sensor readings of a bioprocess. The calculated symptom signal can also compensate for the detected sensor faults [21].

**PRACTICAL APPLICATION**

Predicting process variables that are difficult to measure directly, such as biomass concentration, enables improved monitoring of bioprocesses. However, soft sensors are often subject to inaccuracies and poor precision due to sensor faults in the underlying hardware sensors. This study describes an approach using an ensemble‐based model to achieve fault‐tolerant predictions of process variables, thereby enabling reliable process monitoring.

In addition to fault compensation at the secondary variable level, the correction can be performed directly at the predicted primary variable level. One approach is to constantly update the target variable prediction model during the bioprocess runtime. This concept is called just‐in‐time modeling (JITM). Instead, of a global model, local models are developed for selected time windows [22]. With these local models, sensor drifts can be compensated for, and improved prediction performance for nonlinear processes can be realized. Typically, suitable reference values for calibrating the models are selected from historical datasets. However, ensuring fault‐tolerant predictions requires access to the reference values of the current process. As mentioned above, this real‐time direct access to the target variables is not always available in bioprocesses. A similar approach is to use ensemble‐based models. These models are not developed in real‐time, as is the case with JITM, but offline in advance [23]. Only the combination of models occurs in real‐time. Especially for non‐linear relationships, such a combination of individual linear models can improve prediction performance [24]. Particularly the selection of suitable models is the focus of most studies. The combination in real‐time is done via averaging [25, 26], using the median [27], or by selecting the models depending on the current process parameters [24]. Besides improved prediction performance for non‐linear relations, the above approach can develop a fault‐tolerant soft sensor through a fault‐tolerant combination algorithm.

To enable this real‐time fault‐tolerant combination, approaches from the field of data fusion, also referred to as information fusion or sensor fusion, can be applied [28, 29]. One approach to combine different sensors is to implement a voting structure. On the one hand, an abnormal process can be detected by the deviation of individual sensors [30], which corresponds to a minority‐based voting structure. On the other hand, a majority‐based voting structure can be utilized to exclude redundant measurements or predictions from a set of measurements. Here, the measurements or predictions that give similar outputs would be classified as valid [31]. Also, a decision about the validity of the individual predictions based on thresholds depending on the training data is possible [27]. Another approach is a combination using dynamic weighting parameters for every model. This can be based, for example, on a fusion of several Kalman filters combined through their covariance matrices [32]. Irregular sampling rates and variable measurement delays can also be considered [33]. The combination of such weighting and voting approaches for a fault‐tolerant combination of soft sensor models is promising. It allows addressing different types of faults and their impact on soft sensors. However, only a few approaches from the biotechnological industry are described in the literature that investigates fault tolerance for soft sensors on the level of primary variables [31].

This study investigates the approach of combining independent soft sensor submodels to predict biomass concentration using an ensemble‐based model in a *Pichia pastoris* batch cultivation. For this purpose, three submodels based on independent sensor or actuator information were developed. The first submodel (Base submodel) is based on the relationship between biomass formation and the formation of acids and nitrogen consumption. This is implemented by correlating the consumption of the pH correcting agent, ammonium hydroxide, and biomass concentration. The second submodel (CER submodel) is based on exhaust gas measurements and includes calculating the carbon dioxide emission rate. Here, the formation of CO_2_ is associated with biomass concentration. The third submodel (MIR submodel) uses mid‐infrared measurements to monitor the composition of the fermentation matrix, especially formed metabolites and consumed media components. An ensemble‐based model was developed by combining the three submodels in real‐time. The algorithm sequence can be roughly divided into the following steps: First, a moving window regression is performed to determine the reliability of the current predictions of the submodels. Then, the submodels are penalized based on the comparison by t‐tests with previous predictions. After this penalty, variance‐based weighting is performed. The ensemble‐based model's fault tolerance was tested in simulated and real single and multiple fault scenarios.

## 2 MATERIALS AND METHODS

### 2.1 Strain and preculture conditions

The inoculum of the *P. pastoris* strain (DSMZ 70382) was cultured in three 150‐mL shake flasks containing 50 mL of the medium FM22 supplemented with glycerol as a carbon source. The cultivation takes 70 h at a temperature of 30℃ with a shaking rate of 150 min^−1^. The FM22 was prepared according to Stratton et al. [34]: (NH_4_)_2_SO_4_, 5 g L^−1^; CaSO_4_·2H_2_O, 1 g L^−1^; K_2_SO_4_, 14.3 g L^−1^; KH_2_PO_4_, 42.9 g L^−1^; MgSO_4_·7H_2_O, 11.7 g L^−1^; glycerol, 40 g L^−1^. In addition, 2.0 mL L^−1^ of the trace element solution PTM4 was added to the FM22. The PTM4 solution contained the following: CuSO_4_·5H_2_O, 2 g L^−1^; KI, 0.08 g L^−1^; MnSO_4_·H_2_O, 3 g L^−1^; Na_2_MoO_4_·2H_2_O, 0.2 g L^−1^; H_3_BO_3_, 0.02 g L^−1^; CaSO_4_·2H_2_O, 0.5 g L^−1^; CoCl_2_, 0.5 g L^−1^; ZnCl_2_, 7 g L^−1^; FeSO_4_·H_2_O, 22 g L^−1^; biotin, 0.2 g L^−1^; conc. H_2_SO_4_, 1.0 mL.

### 2.2 Batch cultivation in bioreactor

The three inoculum shake flasks were pooled and transferred into a bioreactor (Biostat Cplus, Satorius AG, Goettingen, Germany) with a working volume of 15 L. The medium of the main culture was again FM22 with PTM4 solution. Ammonium hydroxide was used to control the pH to 5. In addition, pressure (500 mbar), temperature (30℃), and dissolved oxygen (40%) were controlled. A cascade control was used for the dissolved oxygen, with a primary stirring speed (300–600 min^−1^) and then an aeration rate (20–40 L min^−1^) acted as control variables. A total of five batch cultivations were performed.

### 2.3 Bioreactor and sensor systems

**FIGURE 1:**
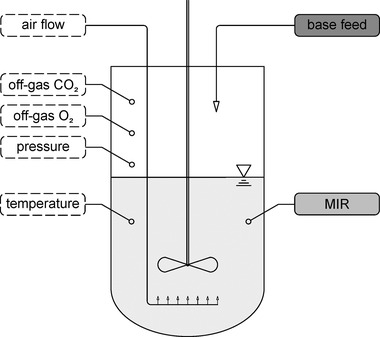
Schematic illustration of the bioreactor. The inputs are assigned to the respective submodels: dashed: CER (carbon dioxide emissions rate) submodel; light gray: MIR (mid‐infrared) submodel; gray: Base submodel

The reactor system had various sensor systems that were relevant for soft sensor development. The sensors can be assigned to the respective soft sensor submodels (see Figure 1). Due to the formation of acids and pH control, a base was added over the process run. The added amount of base was logged and used as input for the Base submodel. The pressure, temperature, airflow, and concentration of CO_2_ and O_2_ in the exhaust gas were determined for the CER submodel. The exhaust gas concentrations were measured with a BlueInOne Sensor (BlueSens gas sensors GmbH, Herten, Germany). For the MIR submodel, the corresponding infrared spectra were recorded with a ReactIR (Mettler‐Toledo GmbH, Gießen, Germany). The instrument was calibrated before each bioprocess and filled with liquid nitrogen for detector cooling every 24 h.

### 2.4 Determination of dry cell weight

The dry cell weight was determined as a reference for the developed soft sensor and submodels. Therefore, samples were taken every 2 h using an autosampler (BaychroMAT, Bayer AG, Leverkusen, Germany). The dry cell weight was determined in triplicates. Therefore, the samples were centrifuged in the previously weighed centrifugation tubes. The supernatant was discarded, and the cell pellet was then dried at 80℃ for 3 days and finally weighed.

### 2.5 Data management

The controller unit of the bioreactor (Biostat Cplus, Satorius AG) was used for process control. SIMATIC SIPAT (Siemens AG, Munich, Germany) was used for managing and logging process and reference data. Preprocessing, data analysis, soft sensor submodel development, and adaptive soft sensor development were realized in MATLAB R2020b (TheMathWorks Inc., Natick, USA).

### 2.6 Development of the soft sensor submodels

#### 2.6.1 Base submodel

The first submodel $y_{Base,\;raw}$ was based on the correlation between the added cumulative base and biomass concentration. The assumption made here is that acids formed are related to biomass concentration. In addition, the function of the base as an additional nitrogen source implies a good correlation with biomass concentration. This linear correlation has already been demonstrated by Brunner et al. [6]. The calibration of the model was performed by least‐squares regression.

#### 2.6.2 Carbon dioxide emission rate submodel

The second submodel $y_{CER,\;raw}$ was developed based on the exhaust gas measurements. The assumption made here is the following: the biomass concentration increases by the amount of CO_2_ formed. The maintenance metabolism can be neglected considering exponential growth. Successful soft sensor developments based on exhaust gas values have already been demonstrated in other publications [5, 6]. In this study, this approach has been implemented as a separate, independent model.

The carbon dioxide emission rate (CER) was calculated to quantify the CO_2_ formed. The airflow rate ${\dot{V}}_{air}$, pressure p, the volume of the liquid $V_{liquid}$, the universal gas constant $R\;(8.314\;\cdot 10^{-2}\;\frac{L\cdot bar}{mol\cdot K})$, and the temperature T and the mole fraction of carbon dioxide $x_{CO2}$ and oxygen $x_{O2}$ in the inlet (indice in) and outlet air (indice out) are required for the calculation [35].

(1) $$\begin{array}{ccc} CER & = & \frac{{\dot{V}}_{air}\cdot p}{V_{liquid}\cdot R\cdot T}\cdot \\ & & \left(\frac{1-x_{O2,\;in}-x_{CO2,in}}{1-x_{O2,out}-x_{CO2,out}}\cdot x_{CO2,out}-x_{CO2,in}\right)\; \end{array}$$

All quantities could be determined directly by hardware sensors, except for the volume of the liquid. Therefore, the starting volume $V_{start}$, the added amount of base $V_{base}$, the added amount of antifoam $V_{antifoam}$, and the volume of the drawn samples $V_{sample}$ were balanced.

(2) $$V_{liquid}=V_{start}\;+V_{base}+V_{antifoam}-V_{sample}$$

CER values were calculated and cumulated to correlate them with biomass concentration using a linear model and the least‐squares method, analogous to the first submodel.

#### 2.6.3 Mid‐infrared submodel

The third submodel $y_{MIR,\;raw}$ was designed using the measurements of a mid‐infrared sensor. The collected spectra contain information about formed metabolites and consumed media components. Since the investigated process is a pure batch process, concentration changes are predominantly associated with the microorganism's metabolism. Therefore, concentration changes can be correlated directly with biomass concentration. In particular, the decrease in sugar concentration can be monitored using mid‐infrared [36] and is reciprocally related to biomass formation. The spectra were smoothed, and the first derivative was calculated using Savitzky‐Golay filter (third‐degree polynomial, window size = 31). Because the spectral data represent a strongly collinear input matrix, partial least‐squares (PLS) regression was used to calibrate the soft sensor model (PLS model). With this technique, stepwise regression can be performed using latent variables with a high covariance to the target variable [37]. For each additional latent variable, the mean square error (MSE) was calculated using cross‐validation. Therefore, the average of the sum of squared deviations between reference values y and estimated values $\hat{y}$ was calculated.

(3) $$MSE=\frac{1}{n}\sum_{i=1}^{n}{\left(y-\hat{y}\right)}^{2}$$

The optimal number of latent variables was determined based on the first local minimum of the MSE.

#### 2.6.4 Submodel validation

A total of five process datasets were available. Three were used for calibrating and validating the submodels (cross‐validation, calibration:validation = 2:1). The remaining two datasets contained sensor faults, so they were used exclusively to test the entire ensemble‐based model. Four latent variables were selected for the PLS model of the MIR submodel. The R^2^ and the root mean square error (RMSE) were used to evaluate the quality of the calibration and validation.

(4) $$RMSE=\sqrt{\frac{1}{n}\sum_{i=1}^{n}{\left(y-\hat{y}\right)}^{2}}$$

### 2.7 Ensemble‐based method for fault‐tolerant submodel combination

#### 2.7.1 Ensemble‐based combination algorithm

**FIGURE 2:**
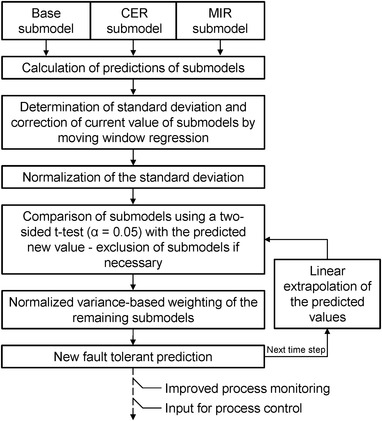
Concept of the ensemble‐based combination algorithm. The length of the moving window for the submodels is 20 time steps (each step is 30 s) and, for the linear extrapolation of the predicted values, the moving window has a size of 10 time steps

Figure 2 illustrates how the three soft sensor submodels (Base, CER, and MIR) are combined into a fault‐tolerant prediction model by the ensemble‐based combination algorithm. The combination is performed without offline reference values. The main intermediate steps of the combination algorithm are described in the following chapters. The algorithm begins by computing the predictions of the three submodels (Base, CER, and MIR) for the current time step.

#### 2.7.2 Moving window regression

The submodels’ reliability was evaluated to combine their predictions in a fault‐tolerant approach. Therefore, one moving window regression per submodel and time step was performed. In this type of linear regression, a linear model is fitted with the predictions of the submodels in a defined time window. Here, the time also acts as the input matrix for the regression. Subsequently, the current submodel prediction $y_{submodel,\;raw}$ can be smoothed by the prediction of the moving window model to a smoothed submodel prediction $y_{submodel}$. The deviations of the submodels to the moving window regression, i.e., the corresponding residuals, can be used in the following as parameters to determine the submodels’ reliability. Therefore, the residuals (as $y-\hat{y}$) of each time window were used in calculating the RMSE (Equation 4). A window size of 10 min (20 time steps) was chosen because a linear increase in biomass concentration could be assumed in these short‐time ranges, and, thus, a sufficient number of submodel predictions were available.

#### 2.7.3 Normalized submodel deviation

The standard deviations of the submodels were normalized to 1 g L^−1^ to assign weight to the predictions of the submodels less in the following two‐step combination only if sensor faults occur. Therefore, the standard deviation per time step, which was calculated by the moving window regression, was divided by the historical mean of the standard deviations of the corresponding submodel. The consequence of this step is that submodels with a particularly small standard deviation are not excluded incorrectly from the fault‐tolerant prediction, and submodels that have a wider spread of values are only weighted less if the standard deviation is abnormally high.

#### 2.7.4 Exclusion of submodels

Next, individual submodels that significantly deviated were excluded from the fault‐tolerant prediction. Therefore, a comparison value was first calculated using a moving window regression of the last ten fault‐tolerant predictions. This comparison value was then compared with the predictions of the respective submodels and their normalized standard deviations using a two‐sided t‐test (α = 0.05). If one or more submodels were significantly different from the comparison value, the submodel was excluded from the current time step from the following steps. This approach is not a classical voting structure, since the voters represent the previous predictions. However, a similar effect can be achieved as with a majority‐based voting structure even if the majority of the sensors would fail in the current time step.

#### 2.7.5 Normalized variance‐based weighting of submodels

Normal distribution was assumed for each submodel to combine the remaining submodels. The smoothed prediction of the submodel served as $y_{submodel}$ and the squared, nominalized standard deviation as variance $\sigma_{submodel}^{2}$. The normal distributions could then be multiplied, allowing for weighting based on the normalized standard deviation. This variance‐based weighting is based on the weighting strategy of the Kalman filter [12]. For all three submodels remaining for combination, the fault‐tolerant prediction $y_{ensembled}$ resulted in Equation (5).

(5) $$\begin{array}{c} y_{ensembled}=\frac{\begin{array}{c} \sigma_{CER}^{2}\cdot \sigma_{MIR}^{2}\cdot y_{Base}+\sigma_{Base}^{2}\cdot \sigma_{MIR}^{2}\cdot y_{CER}\\ +\;\sigma_{Base}^{2}\cdot \sigma_{CER}^{2}\cdot y_{MIR} \end{array}}{\sigma_{CER}^{2}\cdot \sigma_{MIR}^{2}+\sigma_{Base}^{2}\cdot \sigma_{MIR}^{2}+\sigma_{Base}^{2}\cdot \sigma_{CER}^{2}} \end{array}$$

If submodels are excluded, the equation is simplified accordingly. In case no submodel reaches this step, the comparison value of the linear extrapolation of the last ten predictions is used.

Thus, by predicting the ensemble‐based model $y_{ensembled}$, a fault‐tolerant prediction can be made for the current time step. For the next time step, the algorithm described in Figure 2 begins again by computing the predictions of the individual submodels.

#### 2.7.6 Evaluation of ensemble‐based model with simulated and real fault scenarios

To test the ensemble‐based model, a previously used dataset was subjected to simulated fault scenarios. Therefore, three different simulated faults were applied separately: S1, loss of the base logging; S2, increased noise in MIR signal; S3, peak in CO_2_ signal followed by permanent deviation in the CER submodel. The selected scenarios represent three of the typical sensor fault types (S1, complete failure; S2, reduction in precision; S3, bias). In addition, the sequence of the simulated faults was changed to test the fault‐tolerant ensemble‐based model in a multiple fault scenario. Therefore, the permanent deviation of the CER submodel (S3) was transferred before S1 and S2 to obtain the multiple fault scenario S4.

In addition to the simulated fault scenarios, the evaluation was performed in two real fault scenarios (R1, incorrect calibration MIR sensor; R2, data logging failure during base addition, and no refilling of the liquid nitrogen of the MIR sensor). Both process datasets were used exclusively for testing the entire ensemble‐based model.

The RMSE (Equation 4) was calculated for the selected areas (S1–S4 and R1–R2) to evaluate the prediction performance of the ensemble‐based model through statistical parameters. Since only marginal reference values were available for the short ranges, interpolation was performed between the reference values.

## 3 RESULTS AND DISCUSSION

### 3.1 Evaluation of the submodels

**FIGURE 3:**
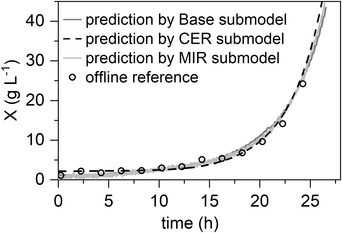
Validation of submodels. An illustration of the three submodels (Base, CER, and MIR) with the corresponding reference values

Figure 3 shows the measured reference values, along with the predictions of the three submodels, in a validation step. A plausible course can be obtained, even in the areas between the reference values. The quality parameters (see Table 1) demonstrate a good prediction performance of the submodels. R^2^ of ≥ 0.98 and RMSE of ≤ 2.4 g L^−1^ are achieved for all submodels. Consequently, three independent soft sensor submodels for biomass concentration prediction were successfully developed. The next step was to combine them using a fault‐tolerant combination algorithm and test the ensemble‐based model in different fault scenarios.

**TABLE 1:** Averaged quality parameters of the submodels. R^2^: Coefficient of determination, RMSE: Root Mean Square Error

	*Base submodel*	*CER submodel*	*MIR submodel*
R^2^	0.98	0.98	0.99
RMSE (g L^−1^)	1.8	1.9	2.4

### 3.2 Evaluation of the ensemble‐based submodel combination

#### 3.2.1 Simulated fault scenarios

**FIGURE 4:**
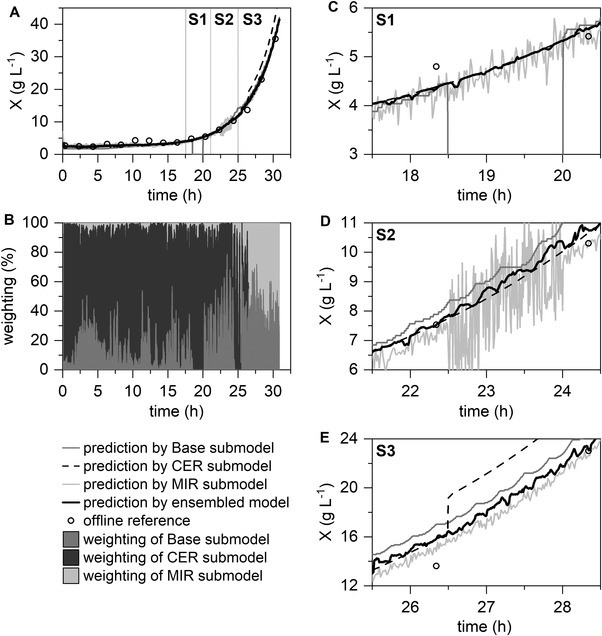
Application of the ensemble‐based model in simulated single fault scenarios. The Base, CER, and MIR submodels are combined to form a final prediction model for biomass concentration predictions (A) with the visualization of the weighting of the submodels (B). Enlarged display of the fault scenarios (C–E): S1, loss of the base logging; S2, increased noise in the MIR signal; S3, peak in CO_2_ signal, as such permanent deviation of the CER submodel

**FIGURE 5:**
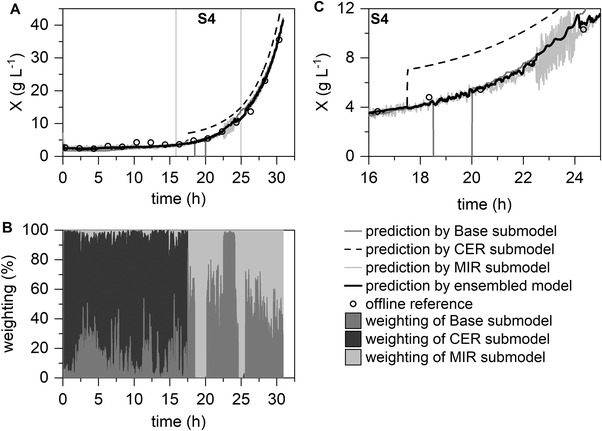
Application of the ensemble‐based model for a simulated multiple fault scenario. The Base, CER, and MIR submodels are combined to form a final prediction model for biomass concentration predictions (A) with the visualization of the weighting of the submodels (B). Enlarged display of the multiple fault scenario (C): S4, differing peak in CO_2_ signal (as such permanent deviation of the CER submodel), loss of base logging, and increased noise in the MIR signal

Figures 4 and 5 illustrate the high reliability of the ensemble‐based model in single and multiple fault scenarios. The weighting plots (Figures 4B and 5B) show that the submodels were successfully penalized in case of base logging failure at hour 18.5 (S1) by exclusion via *t*‐test, as well as increased noise of the MIR submodel at hour 22.5 (S2) via the variance‐based combination. In addition, the exclusion of the CER submodel by t‐test after the permanent deviation (Figure 4 hour 26.5 (S3) and at Figure 5 hour 17 (S4)), and a respective adjustment of the weighting of the other submodels could be realized successfully by the ensemble‐based model. The relatively high degree of noise in the weighting plot can be explained by the high dynamics of the ensemble‐based model through the variance‐based part of the algorithm and the noise of the submodels (Figures 4B and 5B). The repetitive sequence of the algorithm enables dynamic weighting and prevents over‐reliance on a single submodel.

The calculated RMSEs of the submodels and ensemble‐based model are summarized in Table 2. The standard deviation of the reference measurements (dry cell weight) is approximately 0.2 g L^−1^ (data not shown). Thus, in all four cases, the ensemble‐based model represents either the prediction with the lowest RMSE or a comparable RMSE with the most accurate submodel. It is noticeable that in S2, the RMSE of the noisy MIR is still low compared to the other single fault scenarios. This is because the visually very noisy MIR submodels do not produce inaccurate predictions on average. However, due to the strong irregular spread, it is less suitable for control systems and is correctly weighted low by the ensemble‐based model.

**TABLE 2:** RMSE (g L^−1^) of the simulated fault scenarios

*Fault*	*Description*	*RMSE_Base_* (g L^−1^)	*RMSE_CER_* (g L^−1^)	*RMSE_MIR_* (g L^−1^)	*RMSE_ensembled_* (g L^−1^)
S1	Loss of the base logging	4.41	0.24	0.37	0.26
S2	Increased noise in the MIR signal	0.83	0.13	0.98	0.33
S3	Permanent deviation in the CER submodel	1.65	4.68	0.58	0.89
S4	Multiple fault scenario (S1 + S2 + S3)	2.25	2.72	0.62	0.43

This confirms the suitability of a fault‐tolerant soft sensor for simulated faults (complete failure, reduction in precision, and bias) in single and multiple fault scenarios. In addition, in multiple failure scenarios, the majority of the submodels generated faulty predictions at a time (CER + Base submodels or CER + MIR submodels), but a reliable prediction was still possible.

#### 3.2.2 Real fault scenarios

**FIGURE 6:**
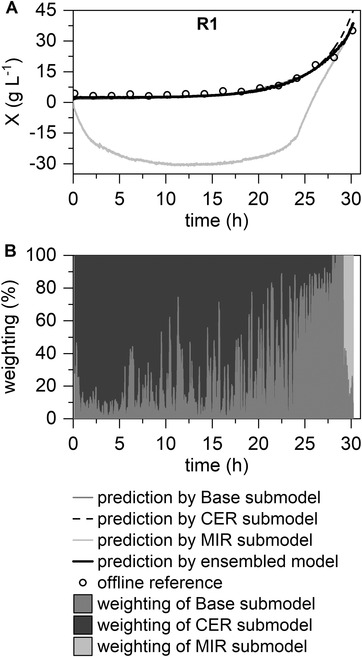
Application of the ensemble‐based model for a real single fault scenario (Fault R1: incorrect calibration MIR sensor). The Base, CER, and MIR submodels are combined to form a final prediction model for biomass concentration predictions (A) with the visualization of the weighting of the submodels (B)

**FIGURE 7:**
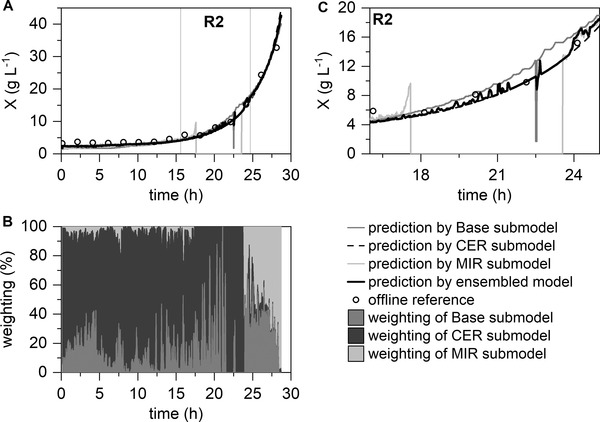
Application of the ensemble‐based model for a real multiple fault scenario. The Base, CER, and MIR submodels are combined to form a final prediction model for biomass concentration predictions (A) with the visualization of the weighting of the submodels (B). Enlarged display of the multiple sensor fault scenario (C): R2, Failure of data logging during base addition, and no refilling of the liquid nitrogen of the MIR sensor

In this section, the developed ensemble‐based model was evaluated using the remaining two datasets. In both datasets, at least one real sensor fault occurred. The impact of the occurred faults is visualized in Figures 6 and 7 for the submodels and ensemble‐based model. As in the previous section, the RMSE of the submodels and ensemble‐based model were calculated (see Table 3).

**TABLE 3:** RMSE (g L^−1^) of the real fault scenarios

*Fault*	*Description*	*RMSE_Base_* (g L^−1^)	*RMSE_CER_* (g L^−1^)	*RMSE_MIR_* (g L^−1^)	*RMSE_ensembled_* (g L^−1^)
R1	Incorrect calibration of the MIR sensor	1.32	1.94	28.75	1.14
R2	Multiple fault scenario (Failure of the base data logging and no refilling of liquid nitrogen of the MIR sensor)	1.35	0.79	7.69	0.70

A distinct deviation of the MIR submodel can be recognized (R1, see Figure 6). The deviation is due to the incorrect calibration of the MIR sensor before the start of the process. Consequently, the recorded MIR spectra significantly differed from the other process runs. However, the MIR submodel was excluded from the algorithm, and a fault‐tolerant prediction was made by the ensemble‐based model (see Figure 6). Finally, toward the end of the process, the MIR submodel again shows better prediction performance and returns to a higher weighting in the ensemble‐based model (> hour 28, Figure 6B). Comparing the RMSEs (see Table 3), the large error of the MIR submodel (28.75 g L^−1^) versus the relatively small error of the ensemble‐based model (1.14 g L^−1^) proves the suitability of the fault‐tolerant prediction in the first real fault scenario. This also demonstrates the ensemble‐based model's potential in generally unsuitable soft sensor models: If the majority of the submodels do not provide a similarly incorrect prediction, the ensemble‐based model can produce reliable predictions.

In the second real fault scenario, two faults occurred simultaneously (see Figure 7). On the one hand, the base data logging failed for a short time. On the other hand, the MIR submodel failed for approximately 6 h due to faulty handling of the measuring instrument. There was also a relatively large deviation between the CER and Base submodels in the R2 area. Despite all these problems, a reliable prediction was guaranteed using the ensemble‐based model. In the weighting plot, it is visible that the MIR submodel was directly excluded by the algorithm using the t‐test when the MIR fault occurred (hour 17.5). The remaining submodels were then given more weight until a larger deviation between the CER and Base submodels occurred. With the following failure of the base logging, an increased weighting of the Base submodel occurs as the base value increases. By crossing the prediction of the ensemble‐based model, the Base submodel is no longer excluded by the *t*‐test. This disappears after a short time due to the untypically large variance of the base signal through the variance‐based weighting of the ensemble‐based model. After 25 h, the ensemble‐based model uses all three submodels. Considering the RMSEs in the fault range R2, excellent prediction performance of the ensemble‐based model with 0.70 g L^−1^ is evident (see Table 3). The CER submodel has comparable prediction performance (0.79 g L^−1^), whereas the other two submodels have higher RMSEs (*RMSE_Base_* = 1.35 g L^−1^; *RMSE_MIR_*. = 7.69 g L^−1^). This demonstrates the reliability of the ensemble‐based model, even in this difficult fault scenario.

In summary, it was demonstrated that the ensemble‐based model is generally comparable to the best submodel in terms of prediction performance. However, more importantly, it is impossible to know in advance which submodel will be the best. In bioprocesses, even small variations in the inoculum or slightly changing strain properties can result in submodels no longer making suitable predictions due to metabolic changes. For this reason, it is critical for a fault‐tolerant prediction to have as many independent submodels available as possible (Base submodel: formation of acids and nitrogen supplier; CER submodel: formation of carbon dioxide; MIR submodel: formation of metabolites, degradation of carbon sources, etc.) and to combine these submodels in a fault‐tolerant way. The ensemble‐based model offers the possibility to automate this combination in real‐time, thereby pooling the information about the submodels.

## 4 CONCLUDING REMARKS

This study demonstrated that three independent submodels (Base, CER, and MIR) could be developed successfully for a *P. pastoris* batch process (R^2^ of ≥ 0.98). Nevertheless, the transferability of the submodels to a following fed‐batch phase may be limited, especially in the case of the MIR model. As shown in other studies, the biomass in the fed‐batch phase could be well modeled using base consumption and off‐gas measurements [6] suggesting transferability of Base and CER submodels. The MIR submodel, in contrast, predicts the biomass concentration indirectly via the decrease of nutrients and sugars in the medium. Due to the addition of a nutrient solution in the fed‐batch phase, this correlation is no longer valid. However, a remedy would be to include the feeding rate to the MIR submodel via a hybrid model approach.

The submodels could be fault‐tolerantly combined in an ensemble‐based model and tested in fault scenarios (Fault types: complete failure, reduction in precision, bias). In the difficult simulated multiple fault scenarios (RMSE = 0.43 g L^−1^) and real multiple fault scenarios (RMSE = 0.70 g L^−1^), the suitability of the ensemble‐based model for a fault‐tolerant biomass prediction could be confirmed. This proves the suitability of ensemble‐based methods for fault‐tolerant prediction at the level of primary variables.

Particularly noteworthy is the ability of the ensemble‐based model to make reliable predictions, even when the majority of submodels make incorrect predictions (shown in the multiple fault scenarios). Therefore, it is only necessary that faulty submodels do not calculate a similarly incorrect value, and an ensemble‐based model's reliable initial value can be calculated. Overall, the ensemble‐based model's reliability increases with each additional independent submodel. For the presented process, for example, an additional mechanistic submodel as described by Jahic et al. [16] could be utilized. They developed a kinetic model based on process knowledge via stoichiometric equations, mass balances, and flow and rate equations. The model is designed for batch and fed‐batch phase. An extension of the presented ensemble‐based model by a mechanistic submodel could make the prediction even more accurate. But even in case of inaccuracies of the mechanistic submodel due to unpredictable variations of the process, a good prediction performance can be expected as well as in the case of sensor faults of data‐driven models.

Consequently, especially the modular extension of the ensemble‐based model by submodels has an excellent potential to further improve the prediction and reliability of target values. Because of the ensemble‐based model's generalizability, its application to other strains and processes is also feasible, provided that at least two independent submodels, preferably three or more, are available.

In addition, the recalibration of soft sensor models based on the weighting plots is feasible. Thus, a submodel that does not experience weighting for a certain period could be maintained. This can also compensate for negative effects on prediction performance caused by changes in strains or variations in process inoculum. To perform such model maintenance [38, 39], intelligent selection of historical data and other submodels can further improve the prediction performance of soft sensors.

## NOMENCLATURE

**Table:** 

CER	$\left[mol\cdot {\left(L\cdot h\right)}^{-1}\right]$	Carbon dioxide emission rate
MSE	$\left[g^{2}\cdot L^{-2}\right]$	Mean square error
p	[bar]	Pressure
R	$\left[L\cdot bar\cdot {\left(mol\cdot K\right)}^{-1}\right]$	Molar gas constant
RMSE	$\left[g\cdot L^{-1}\right]$	Root mean square error
T	[K]	Temperature
V	[L]	Volume
$\dot{V}$	$\left[L\cdot h^{-1}\right]$	Volume flow
x	[−]	Mole fraction
y	$\left[g\cdot L^{-1}\right]$	Biomass concentration
*Greek symbols*		
α	[−]	Significance level
$\sigma^{2}$	$\left[g^{2}\cdot L^{-2}\right]$	Variance
*Indices*		
$CO_{2}$	Carbon dioxide	
ensembled	Ensemble‐based	
in	Inlet	
MIR	Mid‐infrared	
$O_{2}$	Oxygen	
out	Outlet	

## CONFLICT OF INTEREST

The authors have declared no conflicts of interest.

## Data Availability

The data that support the findings of this study are available from the corresponding author upon reasonable request.
